# Predictors for New Native-Vessel Occlusion in Patients with Prior Coronary Bypass Surgery: A Single-Center Retrospective Research

**DOI:** 10.1155/2019/6857232

**Published:** 2019-09-23

**Authors:** Ze Zheng, Zi chao Cheng, Shao ping Wang, Shi ying Li, Jian Wang, Hong yu Peng, Zheng Wu, Wen zheng Li, Yun Lv, Jia yu Tian, Shu juan Cheng, Jing hua Liu

**Affiliations:** Department of Cardiology, Beijing Anzhen Hospital, Capital Medical University, Beijing Institute of Heart, Lung and Blood Vessel Diseases, Beijing, China

## Abstract

**Objectives:**

Chronic total occlusion (CTO) is prevalent in patients with prior coronary artery bypass grafting (CABG). However, data available concerning the prevalence of new-onset CTO of native vessels in patients with prior CABG is limited. Therefore, the objective of the study is to determine predictors for new native-vessel occlusion in patients with prior coronary bypass surgery.

**Methods:**

354 patients with prior CABG receiving follow-up angiography are selected and analyzed in the present study, with clinical and angiographic variables being analyzed by logistic regression to determine the predictors of new native-vessel occlusion.

**Results:**

The overall new occlusion rate was 35.59%, with multiple CTOs (42.06%) being the most prevalent (LAD 24.60% and RCA 18.25%, respectively). Additionally, current smoking (OR: 2.67; 95% CI: 2.60 to 2.74; *p*=0.01), reduced ejection fraction (OR: 1.76; 95% CI: 1.04 to 2.97; *p*=0.04), severe stenosis (OR: 3.65; 95% CI: 2.55 to 5.24; *p*=0.01), and diabetes mellitus (OR: 1.86; 95% CI: 1.34 to 2.97; *p*=0.04) serve as the independent predictors for new native-vessel occlusion.

**Conclusion:**

As to high incidence of postoperative CTO, appropriate revascularization strategies and postoperative management should be taken into careful consideration.

## 1. Introduction

Coronary artery disease (CAD), one of the biggest killers, is responsible for approximately 9 million deaths in 2016 [1]. For most CAD patients, they must receive either coronary artery bypass grafting (CABG) or percutaneous coronary intervention (PCI). In some cases, they must receive the combined treatment of CABG and PCI. Clinically, patients treated by CABG therapy tend to experience more complicated conditions and a higher level of severe coronary artery stenosis because they are selected from the total CAD population based on the complexity of coronary heart disease [2]. The mortality and morbidity advantages of CABG in patients with diabetes have been demonstrated by many research studies [3, 4]. A long-term follow-up study showed that CABG shows a superiority in patients with diabetes and multivessel disease over PCI and medication [5]. Similarly, traditional theory holds that CABG is the gold standard in the treatment of the left main coronary artery (LMCA) disease [6].

Despite survival benefits of successful revascularization being firmly established, bypass grafting has several disadvantages. Meta-analysis has demonstrated that patients treated with CABG will experience higher risks in a cerebrovascular accident [7]. Another disadvantage of CABG, as shown by some research studies, is the progression of primary lesions [8, 9]. It was reported in one study that the prevalence of chronic total occlusion (CTO) among CAD patients with and without prior CABG was 89% and 31%, respectively [10], along with a clinical observation of significant increase of CTO. Another study reported that a bypass graft was associated with new native-vessel disease progression [11]. More importantly, relevant study has shown that native coronary artery CTOs are associated with adverse long-term outcomes [12].

Available evidence has shown that CABG is associated with a high incidence of CTOs, and this may lead to poor prognosis of patients [2].

However, few data regarding the prevalence of new-onset CTOs in native arteries are available, with the relevant risk factors of new-onset CTOs remaining unclear. At present, there is a lack of relevant research on Chinese people, who are the majority of East Asians. Our center, which has the largest number of CABG in China, performs thousands of CABG every year. Therefore, we design this retrospective study to determine incidence and identify independent predictors for postoperative new occlusion in the native vessel.

## 2. Methods

### 2.1. Study Population

This study, following the Helsinki Declaration, was approved by the institutional review board and exempted from written informed consent.

All patients were identified from a retrospective review of the institution's database recording detailed information including baseline characteristics, clinical presentation, angiographic data, and medical and surgical treatment. From Jan 2008 to Jan 2017, a total of 5813 patients undergoing CABG were enrolled in the retrospective single-center study, with the following exclusions (1) insufficient data; (2) patients undergoing concomitant valvular or aortic surgery; (3) patients undergoing emergent or urgent surgery; (4) patients with renal failure requiring dialysis; (5) pregnancy; (6) patients with age less than 18 or more than 75 years; (7) without or follow-up angiography less than 1 or more than 5 years; (8) target lesion revascularization within 1 year; and (9) myocardial infarction in recent 3 months. Follow-up angiography analysis was performed after excluding the subjects above.

### 2.2. Coronary Angiography

A baseline angiography was required for patients within 4 weeks before CABG, and one to five year angiographic follow-up was received for them, along with the performing of diagnostic coronary angiography by experienced and credentialed operators after obtaining written informed consent of angiography. The choice of artery access site (radial or femoral) was made by the interventional physician. Procedures were performed by inserting a 6 or 7 Fr guiding catheter under intravenous administration of 3000–5000 IU heparin, with a requirement of injection of each study vessel with at least 2 orthogonal views. Preoperative coronary angiograms were reviewed by two interventional cardiologists blinded to patient outcomes to determine the presence, location, and length of coronary lesions, along with the conduction of postoperative angiograms by two cardiologists blinded to the baseline interpretation. The major epicardial arteries and major branches (≥2.0 mm in diameter) were assessed, the estimation of which was based on the first onset of angina, prior history of myocardial infarction in the target vessel territory, or comparison with a prior angiogram.

### 2.3. Surgical Procedures and Perioperative Management

Operative reports available were reviewed. SYNTAX (Synergy between Percutaneous Coronary Intervention with Taxus and Cardiac Surgery) score was calculated with each pre-CABG coronary angiogram as the criterion of surgical intervention. After evaluating the severity of coronary lesions, the procedure and selection of grafting were based on the patient's clinical presentation, angiographic features, and conduit availability. Pharmacologic treatment obtained from electronic medical records was determined by cardiovascular comorbidities. Patients received aspirin on the first postoperative day, along with indefinite continuation of low-dose aspirin and statin treatment.

### 2.4. Study Outcomes

Qualified readers evaluated angiograms for initial severity of stenosis, morphologic features, location, and occurrence of lesion progression or occlusion using a side-by-side film review, with the primary study outcome as the occurrence of total occlusion in native coronary arteries.

### 2.5. Definition

CTO is defined as a coronary obstruction with thrombolysis in zero-grade flow of myocardial infarction (TIMI) persisting for at least 3 months. Multivessel coronary disease (MVD) is defined as lesions occurring in the left main coronary artery and or over 50% stenosis of the diameter occurring in at least two main epicardial arteries or the primary branches. Progression was defined as further definite narrowing by 25% [13].

### 2.6. Statistical Analysis

Results for continuous variables were presented as mean ± standard deviation, whereas discrete parameters are expressed as the counts and percentages. Continuous parameters were compared with one-way ANOVA, along with the comparison of discrete data using the Mann–Whitney *U* test. The odd ratio (OR) with 95% CI from a logistic regression model was applied to estimate the risks of particular variables on occlusion of the native vessel. All analyses were performed with the Statistical Package for the Social Science (SPSS) software (version 19; Chicago, IL, USA), which were regarded as statistically significant when the critical value *p* < 0.05.

## 3. Results

### 3.1. Patient Characteristics

**Figure 1:**
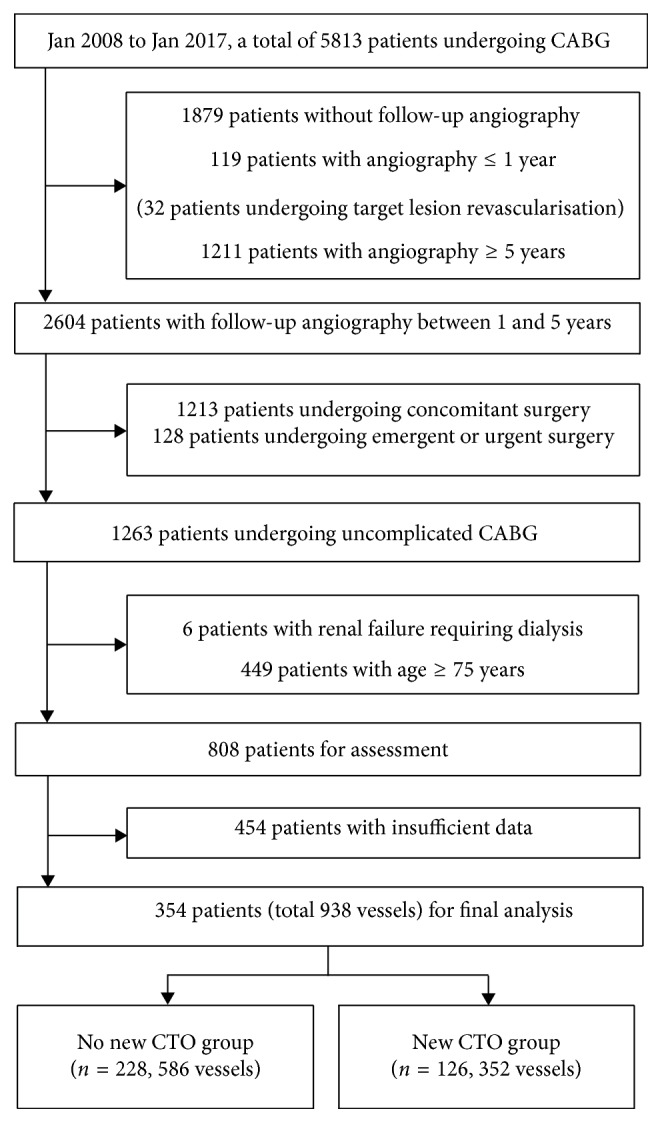
Flow chart of the study.

As shown in Figure 1, of 5813 patients undergoing CABG, a total of 354 patients with 938 vessels met the inclusion criteria and were analyzed subsequently, with baseline characteristics of those 354 patients who underwent follow-up angiography being demonstrated in Table 1. Diabetes mellitus (38.16% vs. 49.20%, *p*=0.044), current smoking (16.67% vs. 47.62%, *p* < 0.001), and lower ejection fraction (59.28% ± 0.07% vs. 56.89% ± 0.1%, *p*=0.010) are more common for patients who suffered postoperative new total occlusion of native arteries (new CTO group) than those without new vessel occlusion during the particular follow-up periods (no new CTO group). In addition, most patients were men with multiple cardiovascular risk factors including hypertension and hypercholesterolemia, however without significant difference between the two groups. Of particular concern were overweight (BMI ≥ 24.0 kg/m^2^) taking up for in nearly 90% of the patients and obesity (BMI ≥ 28.0 kg/m^2^) accounting for approximately one-third of them according to the post-CABG BMI. However, there was no significant difference in the proportion stratified by the BMI category.

### 3.2. Procedural and Angiographic Characteristics

Procedural and lesion characteristics in both groups are presented in Table 2, finding that 35.04% (124 of all cases) patients with at least one CTO had been treated with CABG, and CTOs occurred in 70.62% (250) of cases after CABG (Table 3). Of these, 35.59% (126) suffered from postoperative new total occlusion of native arteries, with preoperative CTO being more likely presented in RCA (14.97%) and most patients having a single postoperative CTO (74.4%), predominantly in the RCA (30.4%). As shown in Table 3, total CTO distribution was as follows: RCA (21.47%); LAD (19.49%); LCX (10.73%); and multiple distributions (18.08%). The overall new occlusion rate was 35.59%, and multiple CTO (42.06%) was most prevalent, followed by LAD (24.60%) and RCA (18.25%). New CTO distribution is summarized in Table 4, showing that among the new CTO patients, the initial lesion was more severe (stenosis ≥ 70%, LAD 90.48% vs. 76.32%; LCX 70.63% vs. 53.95%; RCA 77.78% vs. 57.89%, *p*=0.001). There was no significant difference in the coronary bypass grafting profiles. The mean follow-up was 37 months, without reaching statistical significance compared to the no new CTO (37.52 ± 17.39 vs. 32.65 ± 15.84, *p*=0.175). Although not statistically significant, we found a relatively low proportion of free of symptom and a slightly high revascularization rate (predominantly incomplete revascularization) in patients with new CTO. Moreover, disease progression in native vessels is shown in Table 5 as follows: LM (2.38%); LAD (26.60%); LCX (13.70%); and RCA (18.25%).

### 3.3. Predictors for New Native Coronary Artery Occlusion

Univariate and multivariate analyses were performed to determine the predictors of new native coronary artery occlusion (Table 6). Current smoking (OR: 2.67; 95% CI: 2.60 to 2.74; *p*=0.01), reduced ejection fraction (OR: 1.76; 95% CI: 1.04 to 2.97; *p*=0.04), diabetes mellitus (OR: 1.86; 95% CI: 1.34 to 2.97; *p*=0.04), and initial stenosis ≥ 70%(OR: 3.65; 95% CI: 2.55 to 5.24; *p*=0.01) were associated with an increased risk of new native-vessel occlusion, apart from which, severe stenosis serves as the most powerful predictor among them.

## 4. Discussion

Revascularization of coronary arteries with severe stenosis has reached a consensus in the area of cardiovascular therapeutics and research. For patients with revascularization indications, aggressive revascularization by CABG or PCI is favorable [14]. In clinical practice, patients presenting with complex coronary atherosclerosis, especially multivessel disease, typically are referred for CABG surgery. The preference has also been well supported by published literatures reporting CTO prevalence and treatment. Previous studies have demonstrated that patients with a CTO undergoing PCI and CABG surgery were 4.6–44.98% and 23–40%, respectively, and patients without a CTO were 36–52.9% and 23.2–28%, respectively [15–18]. Surgical revascularization benefits patients with three-vessel or LMCA disease and CTOs including improvement of angina, left ventricular function, and mortality; however, CABG can accelerate stenosis progression of native vessels [16, 19]. Previously, with more attention being paid to graft patency, we observed new native-vessel occlusion after CABG and identified clinical and angiographic predictors for exacerbation of coronary lesions.

Prevalence of CTO is common in patients with prior CABG, and it was reported up to 50% [17]. Similar to previous studies, we found the morbidity of multiple coronary artery stenosis and total occlusion of native arteries in patients post-CABG are considerable in the current study [8, 11, 13]. In this regard, we favor the reasons that patients with complex coronary atherosclerosis were always recommended for surgery and bypass grafts which accelerate stenosis in native vessels [8, 11, 13, 17]. So far, the underlying mechanism remains unknown, and it is speculated that flow competition between the native vessel and graft contributes this progression [8, 11, 13]. In addition, we also found new occlusion which was more common in non-LAD with the findings consistent with the aforementioned studies [8, 11, 13]. Considering the current surgical practice and previous findings that greater incidence of disease progression in segments bypassed with venous grafts, we speculate that it might be associated with the use of venous grafts [19]. However, no significant difference was found between the type of grafts and the new occlusion in this study. Moreover, the present data do not exclude the possibility that local vascular anatomy is the critical pathogenic factor. Regarding the further therapy, patients presenting with recurrent ischemic symptoms, graft failure, or significant progression of native vessels are eligible to undergo revascularization after surgery. It is established that PCI of native coronary arteries is a preferred revascularization strategy for patients with prior CABG, especially those with patent left internal artery bypass grafts. Reasons are given as follows: for the repeat CABG, technical difficulties, increased mortality, and limited symptomatic improvement are major obstacles, and for graft intervention, the increased risk and worse long-term outcomes than native coronary arteries are also a frustrating problem. Even so, previous CABG was associated with the failure of CTO intervention combined with traditionally low success rates and high complication rates [20]. Despite this, the procedural success rate for CTO-PCI has improved over the years with the development of device, technique, and strategy. However, apart from experienced operators, there are several major drawbacks: higher radiation doses, higher volume of contrast agent administered, more severe complications, higher incidence of repeat revascularization, and lower procedural success rates, as compared with non-CTO-PCI [14, 15, 17, 21, 22]. We thus suggest patients with complex multivessel CAD undergo a hybrid approach, such as two-vessel disease with proximal LAD coronary artery occlusion. The hybrid approach is a potential alternative, namely, PCI, for the nonoccluded artery while leaving the CTO vascularized through minithoracotomy approaches, to avoid following weaknesses: surgery-related progression of native coronary lesions, graft stenosis, low primary success rate, and relatively high risk-benefit ratio of CTO-PCI. Furthermore, the hybrid approach facilitates PCI for non-bypassed segments to achieve more complete revascularization by reducing the incidence of new native-vessel occlusion.

Recurrence of effort angina increasing over time was frequently observed in patients with prior CABG. Campeau et al. have revealed the proportion of patients without symptoms after the procedure decreased from 72% to 37% in 10 years of follow-up [23]. In the present study, we found a higher proportion of patients with new occlusion complained of recurrent angina which has driven more repeat percutaneous revascularization of native coronary arteries, with accompanying majority incomplete revascularization. Correspondingly, greater follow-up major adverse cardiovascular event (MACE) rates were also found in these patients. According to a previous study, there were pathological differences in CTO patients with and without CABG [24]. CTOs with CABG have extensive calcification which has largely been attributed to blood stasis and low shear stress resulting from competitive flow between the native and bypass graft [25]. It has also been confirmed that calcification makes CTO-PCI difficult, and the success rate for CTO with prior CABG is significantly lower than those without CABG [25, 26]. Therefore, it is difficult or impossible to achieve complete revascularization. Incomplete revascularization results in persistent left ventricular dysfunction which in turn leads to a worse outcome on follow-up (higher mortality) [20, 27].

Previously, a study has reported clinical and angiographic predictors for native coronary vessel occlusion including bypass graft for non-LAD arteries and graft occlusion and no use of aspirin for LADs [11]. Our data suggest that independent risk factors for lesion occlusion also included diabetes, current smoking, initial lesion severity, and lower ejection fraction. The correlation of initial lesion severity with disease worsening is similar to the finding that progression of atherosclerosis with significant stenosis occurs 10 times as frequently in bypassed arteries as in non-bypassed arteries [13]. In addition, CTO-PCI was frequently performed among patients with prior CABG with lower technical success rates compared to patients without prior CABG [25]. In this regard, minimally diseased coronary arteries were recommended not to be bypassed [13]. In conclusion, our data show few predictors of occlusion in native arteries after CABG. Of note, these indicate the clinical importance of risk factor management against subsequent native-vessel occlusion in the postoperative period. It is worth noting that these predictors are also risk factors for general CAD patients except lower ejection fraction. Patients with these predictors tend to have more complexity of coronary heart diseases. For patients with these predictors, appropriate revascularization strategies such as a hybrid approach should be taken into careful consideration to reduce new native-vessel occlusion. Further study is needed to explore the differences of predictors for new native-vessel occlusion between the average CAD patient population and patients with prior CABG.

### 4.1. Study Limitation

There were several limitations to our study. First, it is a retrospective design. Second, the surgical procedures were performed in a single institution but not by a single surgeon. Our institution does not routinely perform coronary angiography on all patients who have undergone CABG. This means that the study has a bias in the cohort. A prospective study is needed to further explore this issue. An additional limitation of the study is the heterogeneity bias for operator-related and procedure-related factors.

## Figures and Tables

**Table 1:** Baseline patient characteristics.

Variables	All (*N* = 354)	No new CTO (*n* = 228)	New CTO (*n* = 126)	*p* value
Age (yrs.)	61.71 ± 9.47	62.75 ± 7.93	61.80 ± 8.38	0.492
＜65 (%)	212 (59.89%)	136 (59.64%)	76 (60.31%)	
65 to ＜75 (%)	142 (40.11%)	92 (40.35%)	50 (39.68%)	
Sex				0.694
Male	264 (74.58%)	169 (74.12%)	95 (75.40%)	
Female	90 (25.42%)	59 (25.88%)	31 (24.60%)	
BMI (kg/m^2^)	26.57 ± 3.88	27.06 ± 2.97	26.59 ± 3.48	0.397
≤18.4	0	0	0	
18.5–23.9	54 (15.25%)	30 (13.16%)	24 (19.05%)	
24.0–27.9	186 (52.54%)	125 (54.82%)	61 (48.41%)	
≥28	112 (31.64%)	72 (31.58%)	40 (31.75%)	
Hypertension	248 (70.06%)	158 (69.30%)	90 (71.43%)	0.532
Hypercholesterolemia	206 (58.19%)	129 (56.59%)	77 (61.11%)	0.217
Diabetes mellitus	140 (39.55%)	87 (38.16%)	62 (49.20%)	0.044
Diet-controlled	10 (7.14%)	6 (6.90%)	4 (6.45%)	
Tablet-controlled	64 (45.71%)	39 (44.82%)	30 (48.39%)	
Insulin treatment	66 (47.15%)	42 (48.28%)	28 (45.16%)	
Smoker				*p* < 0.001
Never	112 (31.64%)	79 (34.64%)	33 (26.19%)	
Former	144 (40.68%)	111 (48.68%)	33 (26.19%)	
Current	98 (27.68%)	38 (16.67%)	60 (47.62%)	
Prior MI	90 (25.42%)	58 (25.44%)	32 (25.40%)	0.990
Prior PCI	68 (19.21%)	47 (20.61%)	21 (16.67%)	0.178
Prior cardiac surgery	2 (0.57%)	2 (0.88%)	0 (0.00%)	0.525
Prior heart failure	36 (10.17%)	21 (9.21%)	15 (11.91%)	0.237
Peripheral vascular disease	74 (20.90%)	45 (19.74%)	29 (23.02%)	0.279
	70 (19.77%)	46 (20.18%)	24 (19.05%)	0.704
Cerebrovascular disease	24 (6.78%)	14 (6.14%)	10 (7.94%)	0.337
Family history of CAD	80 (22.60%)	52 (22.81%)	28 (22.22%)	0.345
Atrial fibrillation/flutter	36 (10.17%)	18 (7.90%)	18 (14.29%)	0.480
				0.104
Presentation of ACS	12 (3.39%)	8 (3.51%)	4 (3.17%)	
CCS class of angina	218 (61.58%)	145 (63.60%)	73 (57.94%)	
Grade 1	110 (31.07%)	68 (29.82%)	42 (33.33%)	
Grade 2	14 (3.69%)	8 (3.51%)	6 (2.78%)	
Grade 3	58.43 ± 0.08%	59.28% ± 0.07%	56.89% ± 0.1%	0.010
Grade 4	11 (3.10%)	3 (1.32%)	8 (6.35%)	
Ejection fraction (%)	31 (8.76%)	14 (6.14%)	17 (13.49%)	
≤40%	312 (88.14%)	211 (92.54%)	101 (80.16%)	
40%–50%				0.404
≥50%	150 (42.37%)	101 (44.30%)	49 (38.89%)	
Left ventricular grade	164 (46.33%)	100 (43.86%)	64 (50.79%)	
Class I	38 (10.73%)	26 (11.40%)	12 (9.52%)	
Class II	2 (0.57%)	2 (0.88%)	0 (0.00%)	
Class III	77.6 ± 20.47	73.93 ± 17.94	79.09 ± 21.30	0.079
Class IV	87.2 ± 22.49	90.5 ± 20.11	85.89 ± 23.33	0.172
Creatinine (*μ*mol/l)	38 (10.73%)	51.19 ± 7.03	46.46 ± 11.85	0.484
GFR (ml/min/1.73 m^2^)	2.47 ± 4.27	3.01 ± 4.40	2.26 ± 4.22	0.700
GFR < 60 ml/min/1.73 m^2^				
Hs-CRP (mg/L)				

**Table 2:** Procedural and angiographic characteristics of patients with or without a new CTO.

Variables	No new CTO (*n* = 228)	New CTO (*n* = 126)	*p* value
Preoperative angiogram			
Vessel stenosis at baseline			0.001
Moderate (40%–69%)			
LAD	18 (7.89%)	7 (5.56%)	
LCX	18 (7.89%)	6 (4.76%)	
RCA	22 (9.65%)	9 (7.14%)	
Severe (≥70%)			
LAD	174 (76.32%)	114 (90.48%)	
LCX	123 (53.95%)	89 (70.63%)	
RCA	132 (57.89%)	98 (77.78%)	
Coronary lesion category			0.775
Single-vessel disease	18 (7.89%)	8 (6.35%)	
Double-vessel disease	62 (27.19%)	31 (24.60%)	
Triple-vessel disease	116 (64.47%)	87 (69.05%)	
Left main involvement	102 (44.74%)	48 (38.10%)	0.508
Total no. of patients with ≥1 CTO	83 (36.40%)	44 (34.92%)	0.416
Total of CTO vessel			0.984
LAD	23 (10.09%)	15 (11.91%)	
LCX	15 (6.58%)	7 (5.56%)	
RCA	35 (15.35%)	18 (14.29%)	
Multivessel	8 (3.51%)	3 (2.38%)	
Mean no. of bypass grafts	3.0 ± 0.63	3.06 ± 0.75	0.599
Types of graft			0.460
Internal mammary artery	183 (80.26%)	107 (84.92%)	
Saphenous vein	2.22 ± 0.64	2.25 ± 0.73	
Graft patency			0.522
LIMA	190 (83.33%)	109 (86. 51%)	
SVG-D	195 (85.53%)	96 (76.19%)	
SVG-LCX/OM	185 (81.14%)	93 (73.81%)	
SVG-RCA/PDA	163 (71.49%)	84 (66.67%)	
Postoperative medication			0.902
Aspirin	215 (94.30%)	118 (93.65%)	
Statin	185 (81.14%)	98 (77.78%)	
Follow-up time (Mths)	32.65 ± 15.84	37.52 ± 17.39	0.175
Free of symptom	123 (53.94%)	56 (44.44%)	0.087
Incomplete revascularization	112 (49.12%)	70 (55.56%)	0.125

**Table 3:** Characteristics of lesion distribution. Preoperative and postoperative analyses of CTO distribution.

Vessel	Pre-CABG (*n* = 12 35.04%)	Post-CABG (*n* = 250 70.62%)
LM	0 (0%)	3 (0.85%)
LAD	38 (10.73%)	69 (19.47%)
LCX	22 (6.22%)	38 (10.73%)
RCA	53 (14.97%)	76 (21.47%)
Multi vessels	11 (3.12%)	64 (18.08%)

**Table 4:** Distribution of new CTO in native vessels.

Vessel	New CTO in native vessels (*n* = 126)
LM	3 (2.38%)
LAD	31 (24.6%)
LCX	16 (12.7%)
RCA	23 (18.25%)
Multivessels	53 (42.06%)

**Table 5:** Disease progression in native vessels.

Vessel	Overall (*N* = 938)	No new CTO (*n* = 586)	New CTO (*n* = 352)
LM	22 (2.38%)	12 (2%)	3 (0.85%)
LAD	250 (26.6%)	42 (7.11%)	69 (19.49%)
LCX	129 (13.7%)	17 (2.97%)	38 (10.73%)
RCA	171 (18.25%)	37 (6.53%)	40 (11.47%)

**Table 6:** Predictors for new native-vessel occlusion.

Variables	Univariate analysis			Multivariate analysis		
	OR	95% CI	*p* value	OR	95% CI	*p* value
DM	1.57	1.01–2.43	0.044	1.86	1.34–2.97	0.04
Current smoking	2.95	1.51–5.75	0.001	2.67	2.60–2.74	0.01
Ejection fraction	2.16	1.19–3.92	0.010	1.76	1.04–2.97	0.04
Creatinine	1.35	0.91–1.97	0.079			
Severe stenosis (≥70%)	3.85	2.16–6.87	0.001	3.65	2.55–5.24	0.01

## Data Availability

The data of this study can be obtained from the corresponding author only with a reasonable request. The data are not publicly available because the availability of these clinical data was made under ethical license conditions applied for this study, which contained information that could compromise the privacy of research participants.
